# Alternative Splicing and DNA Damage Response in Plants

**DOI:** 10.3389/fpls.2020.00091

**Published:** 2020-02-19

**Authors:** Barbara Anna Nimeth, Stefan Riegler, Maria Kalyna

**Affiliations:** Department of Applied Genetics and Cell Biology, BOKU—University of Natural Resources and Life Sciences, Vienna, Austria

**Keywords:** alternative splicing, DNA repair, DNA damage response, Arabidopsis, plant, stress, splicing factor

## Abstract

Plants are exposed to a variety of abiotic and biotic stresses that may result in DNA damage. Endogenous processes - such as DNA replication, DNA recombination, respiration, or photosynthesis - are also a threat to DNA integrity. It is therefore essential to understand the strategies plants have developed for DNA damage detection, signaling, and repair. Alternative splicing (AS) is a key post-transcriptional process with a role in regulation of gene expression. Recent studies demonstrate that the majority of intron-containing genes in plants are alternatively spliced, highlighting the importance of AS in plant development and stress response. Not only does AS ensure a versatile proteome and influence the abundance and availability of proteins greatly, it has also emerged as an important player in the DNA damage response (DDR) in animals. Despite extensive studies of DDR carried out in plants, its regulation at the level of AS has not been comprehensively addressed. Here, we provide some insights into the interplay between AS and DDR in plants.

## DNA Damage Response in Plants

The genomic integrity of living cells is perpetually challenged by a variety of environmental and internal cellular factors. Environmental stresses, such as drought, salinity, ultraviolet (UV), ionizing radiation, xenobiotic toxicity, heavy metals, and mutagenic chemicals damage DNA and affect its stability (Hu et al., 2016; Nisa et al., 2019). Cellular replication, recombination errors, and reactive oxygen species resulting as a byproduct of metabolism also cause DNA damage. A cell's reaction to genotoxic stress, referred to as DNA damage response (DDR), starts with cell cycle arrest and, in the case of plants, endoreplication (De Veylder et al., 2011). To ensure the repair of a variety of different types of DNA lesions, several DNA repair mechanisms are active and constitute the DNA repair phase of DDR. Should the repair of DNA damage not be sufficient, programmed cell death eliminates the damaged cell and ensures homeostasis (Manova and Gruszka, 2015; Kim et al., 2019). Due to their sessile nature, plants find themselves at increased risk to detrimental environmental factors. It has also been shown that light and temperature conditions affect DNA repair mechanisms such as homologous recombination and photoreactivation (Li et al., 2002; Boyko et al., 2005).

The repair of UV-induced lesions by photoreactivation appears to be an ancient conserved DNA damage repair mechanism. It relies on the activity of photolyase, utilizing the energy of UV-A or blue light to reverse UV damage in the DNA (Manova and Gruszka, 2015; Kavakli et al., 2017; Zhang et al., 2017a). Another mechanism of UV damage repair is nucleotide excision repair (NER), which identifies, removes, and repairs the damaged base(s) using the other DNA strands as a template. In addition to UV lesions, NER repairs bulky adducts that change the DNA conformation. Global genomic repair (GGR) and transcription-coupled repair (TCR), although differing in their mode of damage recognition, share similarities in their mechanisms of action (Hanawalt, 2002). The DNA glycosylases, which initiate base excision repair (BER) at damaged sites, facilitate the repair of a variety of DNA lesions (Wallace, 2014). There is evidence for BER being active in chloroplasts to counter the effects of reactive oxygen species production during photosynthesis (Gutman and Niyogi, 2009). The mismatch repair (MMR) pathway is responsible for the repair of replication errors, such as mismatches and indels, UV, and oxidative damage (Li et al., 2016; Liu et al., 2017; Belfield et al., 2018). Double-strand breaks (DSBs) are repaired *via* non-homologous end joining (NHEJ) and homologous recombination (HR). While HR requires homologous sequences to ensure efficient repair, NHEJ joins DSBs without considering sequence context and is, thus, an error prone mechanism, which can result in mutations and DNA changes (Manova and Gruszka, 2015).

Two protein kinases, ATM (ATAXIA-TELANGIECTASIA MUTATED) and ATR (ATAXIA TELANGIECTASIA-MUTATED AND RAD3-RELATED), initiate eukaryotic DDR. Once activated, they signal *via* checkpoint kinases 1 and 2 (CHK1 and CHK2), respectively. Human homologs of CHK1 and CHK2 activate p53, which in turn controls cell cycle arrest, DNA damage repair, and programmed cell death. While the downstream processes of ATM, ATR, and p53 have been studied extensively, data on their upstream activation and regulation remains scarce. Neither orthologs of CHK1 and CHK2, nor of p53, have been identified in plants so far. However, a functional homolog of p53, SUPPRESSOR OF GAMMA RESPONSE 1 (SOG1), transcriptionally regulating DDR downstream of ATM and ATR was found (Preuss and Britt, 2003; Yoshiyama et al., 2009; Yoshiyama, 2016). Indeed, SOG1 was identified as a master regulator transcription factor of the plant DDR, influencing expression of genes related to the cell cycle and DNA repair (Ogita et al., 2018). About 300 direct targets of SOG1 were identified, including transcription factors, DNA repair genes, and regulators of the cell cycle (Bourbousse et al., 2018).

A recent research update highlights the growing interest in DDR in plants but also serves to show that a role for alternative splicing (AS) remains to be established (Gimenez and Manzano-Agugliaro, 2017).

## Overview of Alternative Splicing

Most messenger RNAs in higher eukaryotes are synthesized as precursors, which contain intervening sequences, known as introns. To provide a template for protein synthesis, messenger RNA (mRNA) introns have to be removed and exons joined in a process termed pre-mRNA splicing. However, exons and introns or their parts can be differentially included in mRNA by AS. AS produces transcript and protein variants from a single gene with different fates and functions, and is a fundamental aspect of RNA biology that has a key role in our understanding of gene expression regulation. Up to 95% of human and 70% of plant multi-exonic genes are alternatively spliced (Pan et al., 2008; Wang et al., 2008; Marquez et al., 2012; Chamala et al., 2015; Zhang et al., 2017b). Further studies report that about 50% of the genes in soybeans, 46% in rice, 40% in maize, and over 60% in tomatoes and barley undergo AS (Thatcher et al., 2014; Chamala et al., 2015; Clark et al., 2019; Rapazote-Flores et al., 2019), emphasizing its importance in crop plant development and environmental response. AS has a broad role in many aspects of plant biology, but its role in responding to DNA damage is mostly unknown and requires further investigation.

Pre-mRNA splicing requires the core splicing signals, which consist of the 5' and 3' splice sites and a branch site (Wang and Burge, 2008). However, multiple additional features, such as intronic and exonic splicing regulatory *cis*-elements (splicing enhancers and silencers), length of introns and exons, and differential guanine-cytosine content between exons and introns, affect the recognition and selection of the core splicing signals (Braunschweig et al., 2013). The secondary structure of the pre-mRNA can alter access to splicing signals and binding sites for splicing factors (SFs) or change the distance between these elements (Shepard and Hertel, 2008). Differential DNA methylation, histone modifications, and nucleosome positioning modulate RNA polymerase II elongation speed and recruitment of SFs, thus also resulting in alternative splice site selection [for a recent review see (Jabre et al., 2019)].

Common types of AS events include exon skipping, usage of alternative 5' and 3' splice sites, mutually exclusive exons, and intron retention. Exon skipping is the predominant event in animals, whereas it is infrequent in plants (Marquez et al., 2012; Braunschweig et al., 2013). Intron retention is widespread both in plants and animals (Marquez et al., 2012; Braunschweig et al., 2014). Interestingly, intron retention transcripts are often not substrates for nonsense-mediated mRNA decay due to their nuclear localization (James et al., 2012; Kalyna et al., 2012; Leviatan et al., 2013; Gohring et al., 2014). Retention of introns may regulate protein abundance during developmental transitions and in response to stress (including DNA damage). When transcripts with retained introns are recognized as incompletely processed they remain in the nucleus until a change in the cellular environment results in post-transcriptional splicing (Yap et al., 2012; Boothby et al., 2013; Boutz et al., 2015; Brown et al., 2015). Microexons (ultra-short exons of 3-30 nucleotides) found in hundreds of animal genes, and recently identified exitrons (alternatively spliced internal regions of protein-coding exons), which occur in ~7% of Arabidopsis and 4% of human protein-coding genes, complement the repertoire of AS events (Marquez et al., 2012; Irimia et al., 2014; Marquez et al., 2015; Staiger and Simpson, 2015; Sibley et al., 2016; Ustianenko et al., 2017; Zhang et al., 2017b).

Hundreds of proteins participate in the splicing process (Chen and Moore, 2015). However, the modulation of splice site recognition is mainly governed by two families of SFs - serine/arginine-rich (SR) proteins and heterogeneous nuclear ribonucleoproteins (hnRNPs) - through binding to regulatory *cis*-elements in the pre-mRNA (Barta et al., 2010; Manley and Krainer, 2010; Yeap et al., 2014; Howard and Sanford, 2015). SR proteins and hnRNPs act as activators and repressors of splice site selection, respectively, however, the effect often depends on their binding position. Expression levels, localization, and post-translational modifications (PTMs) (phosphorylation, acetylation, ubiquitination, and sumoylation) of SFs in a particular cell are one of the components of the splicing code, which governs the AS outcomes (Barash et al., 2010; Baralle and Baralle, 2018). Interestingly, SR proteins and hnRNPs participate in multiple cellular processes, such as mRNA export, RNA stability and quality control, and translation.

## Alternative Splicing and DNA Damage Response, Insights From Studies in Animals

It is becoming clear that RNA-binding proteins and AS are important in DDR. One of the first pieces of evidence that SFs may have a role in DDR came from a study which demonstrated that the depletion of a canonical human SR protein, SRSF1 (SF2/ASF), resulted in increased DSB formation and genome instability (Li and Manley, 2005). Several studies in animals have unexpectedly identified SFs and other RNA processing proteins associated with response to irradiation and DNA damaging chemicals. For example, genome-wide siRNA knockdown of multiple genes have shown that splicing and RNA processing factors are the most enriched functional category within factors whose depletion mediates DNA damage (Paulsen et al., 2009; Lackner et al., 2011). Studies of individual SFs, including SR proteins, have demonstrated changes in their expression levels, AS profiles, phosphorylation state, and subcellular distribution in response to DNA damage (Matsuoka et al., 2007; Busa et al., 2010; Sakashita and Endo, 2010; Ip et al., 2011; Adamson et al., 2012; Leva et al., 2012). The importance of AS and splicing factors in DDR in animals has been reviewed extensively (Naro et al., 2015; Shkreta and Chabot, 2015; Giono et al., 2016; Kai, 2016; Mikolaskova et al., 2018).

**Figure 1:**
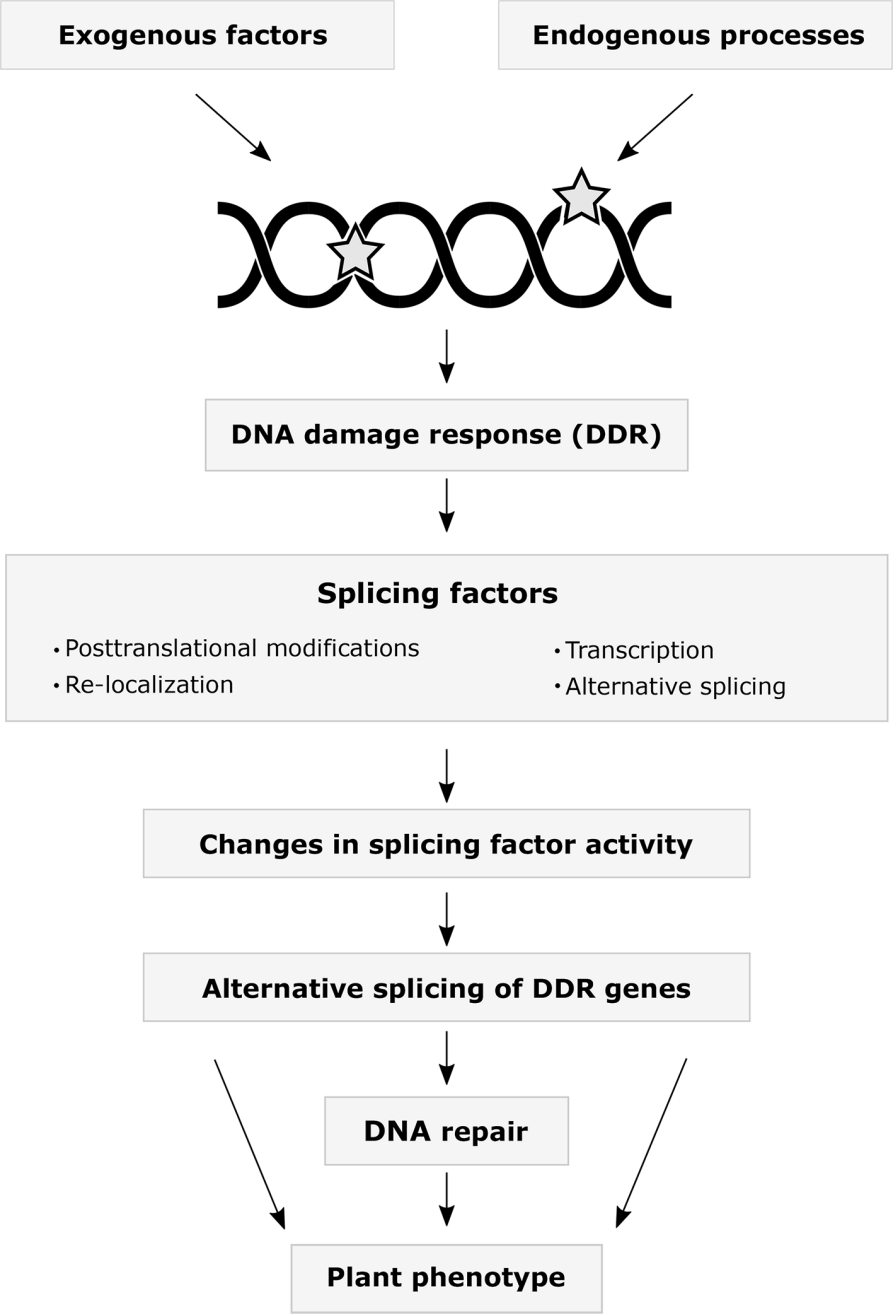
The interplay between the DNA damage response and alternative splicing. A variety of exogenous environmental stress factors and endogenous cellular processes may result in DNA damage. Numerous studies on animals have demonstrated that splicing factors change their expression levels, alternative splicing patterns, post-translational modification states, and subcellular localization in response to DNA damage. Altered expression and activities of splicing factors may regulate DNA repair by modulating alternative splicing of DDR genes. Current data indicates that many plant DDR genes undergo alternative splicing. Which plant splicing factors are involved in the DDR, how they are regulated, what are their target genes, and how the splicing changes are translated into the plant phenotype remains to be addressed in the future.

The interplay between DDR and AS occurs at multiple levels (**Figure 1**). One of the most rapid responses to stress and DNA damage is the change in activity of already translated proteins by PTMs. Multiple SFs have been identified in DDR-regulated phosphoproteomes (Bennetzen et al., 2010; Bensimon et al., 2010; Beli et al., 2012). The kinases ATM and ATR are directly activated by DNA lesions and phosphorylate hundreds of proteins in response to ionizing radiation, including several hnRNPs and SR proteins (Matsuoka et al., 2007). Studies using the treatment of mammalian cells with several genotoxic agents revealed reduced SR protein phosphorylation levels affecting their accumulation in nuclear granules. These studies also found differential AS of genes involved in DNA repair, cell cycle control, and apoptosis (Bennetzen et al., 2010; Leva et al., 2012; Shkreta et al., 2016). Remarkably, detained introns, a recently identified subgroup of retained introns, are enriched in genes involved in DDR. Moreover, DNA damage and the activity of certain Clk kinases, which maintain the hyperphosphorylated status of SR proteins, can modulate splicing of detained introns (Boutz et al., 2015). Changes in the activity of SR proteins also have been associated with their acetylation state in response to cisplatin-induced DNA damage (Edmond et al., 2011; Nakka et al., 2015). Interestingly, acetyltransferases can indirectly impact the translocation of SR proteins *via* the modification of SR protein kinases (Edmond et al., 2011). Recent studies also demonstrated the acetylation of hnRNPs in response to DNA damage (Magni et al., 2019; Siam et al., 2019). Ubiquitination, besides its regulatory activity during spliceosome assembly, affects SFs upon DNA damage (Lu and Legerski, 2007). Genotoxic agents cause deubiquitylation and sumoylation of hnRNPs (Vassileva and Matunis, 2004).

As localization and shuttling of SFs is highly dependent on their phosphorylation state, it is not surprising that DNA damage-induced nuclear translocation of SR protein kinases results in the hyperphosphorylation and subsequent nuclear accumulation of certain SR proteins (Edmond et al., 2011). UV irradiation also affects the redistribution of SFs into the cytoplasm, therefore impacting AS (van der Houven van Oordt et al., 2000; Llorian et al., 2005; Guil et al., 2006). The DNA damage-induced re-localization of SFs appears to be dependent on cell type and genotoxic treatment (Tissier et al., 2010; Wong et al., 2013).

In plants, members of different Arabidopsis SR protein sub-families localize into distinct populations of nuclear speckles (Lorkovic et al., 2008), with their localization dependent on their phosphorylation status (Ali et al., 2003; Tillemans et al., 2005). Different classes of kinases (such as SR protein kinases, PRP4 kinases, Cdc2-like or LAMMER-type kinases, and mitogen-activated protein kinases) phosphorylate plant SFs, including SR proteins and hnRNPs (Golovkin and Reddy, 1999; Savaldi-Goldstein et al., 2000; Feilner et al., 2005; de la Fuente van Bentem et al., 2006; de la Fuente van Bentem et al., 2008; Kanno et al., 2018), suggesting that DNA damage in plants could lead to altered SF activities and changes in AS. However, to which extent this occurs, which SFs are affected and the roles of different PTMs remain the subject of further studies.

In addition to the post-translational regulation of SFs during DDR, their activity can be altered by changes in their AS. Studies in animal cells have illustrated the impact DNA damage has on the AS of SF genes (Solier et al., 2010; Ip et al., 2011; Leva et al., 2012). Munoz and colleagues describe a mechanism by which AS is regulated during DDR (Munoz et al., 2009; Munoz et al., 2017). The hyperphosphorylation of the C-terminal domain of RNA polymerase II (RNAPII) is associated with a decrease in RNAPII elongation speed. This slowing down of RNAPII favors the selection of weaker splice sites as the time window for their recognition by the splicing machinery is extended before stronger downstream sites are synthesized. The hyperphosphorylation and slowdown of RNAPII in response to UV exposure leads to differential exon skipping events in multiple genes associated with apoptosis, cell cycle, and cancer (Munoz et al., 2009; Munoz et al., 2017). These findings raise questions regarding the mechanisms and PTMs affecting RNAPII elongation speed and the subsequent changes in splicing outcomes during DDR in plants. Which plant SFs are alternatively spliced during DDR, how their transcript isoforms differ in their function, and how their AS influences DDR itself also remains to be addressed in the future.

## Alternative Splicing, a New Player in the Plant DNA Damage Response?

Despite extensive studies of DDR and AS in animals, comparatively little is known about this relationship in plants. The PubMed search with the terms “Splicing” and “DNA damage” or “DNA repair” returns a handful of papers in the plant field, which is in stark contrast to about 700 non-plant papers. The first papers describing AS of the Arabidopsis DNA damage/repair gene At-FPG/At-MMH DNA glycosylase were published about 20 years ago (Ohtsubo et al., 1998; Murphy and Gao, 2001). Since then, several key DNA repair genes have been reported to undergo AS, supporting the importance of AS in DDR in plants (**Table 1**). For example, genes encoding At-RAD1/UHV1 (homologous to yeast RAD1 and human XPF DNA repair endonuclease) and AtPOLK polymerase generate AS isoforms in a tissue-specific pattern (Vonarx et al., 2002; Garcia-Ortiz et al., 2004; Garcia-Ortiz et al., 2007). Two Arabidopsis translesion synthesis DNA polymerases, AtREV and AtPOLH, are regulated by AS, and complementation analysis of AtPOLH AS isoforms in *Rad30*-deficient yeast showed that the AtPOLH C-terminus is required for functional activity (Santiago et al., 2009). Several studies also reveal differential AS in DNA repair genes in crop plants, such as rice class II DNA photolyase (Hirouchi et al., 2003), endonuclease OsMUS81 (Mimida et al., 2007), and checkpoint protein OsRad9 (Li et al., 2017).

**Table 1:** Overview of alternative splicing in genes involved in DNA damage response.

	Gene name	Gene ID	Alternative splicing			Gene name	Gene ID	Alternative splicing	
			AtRTD2^1^	Reference				AtRTD2^1^	Reference
**A**	Base excision repair (BER)				**C**	Homologous recombination (HR)			
	OGG1	At1g21710	+			MRE11	At5g54260	+	
	FPG	At1g52500	+	2,3		RAD50	At2g31970	+	
	NTH1	At2g31450	+			NBS1	At3g02680	+	
	NTH2	At1g05900	+			COM1	At3g52115	+	
	DME	At5g04560	+			RECQ4A	At1g10930	+	
	ROS1	At2g36490	+			RAD51	At5g20850	+	
	UNG	At3g18630	–			RAD51B	At2g28560	+	
	DML3	At4g34060	+			RAD51C	At2g45280	+	7
	MBD4L	At3g07930	+	4		RAD51D	At1g07745	+	
	ARP	At2g41460	+			XRCC2	At5g64520	+	
	APE1L	At3g48425	+			XRCC3	At5g54750	+	7
	APE2	At4g36050	+			BRCA2A	At4g00020	+	
	ZDP	At3g14890	+			BRCA2B	At5g01630	–	
	TDP1	At5g15170	+			RAD54	At3g19210	+	
	XRCC1	At1g80420	+			SRS2	At4g25120	+	
	SAV6	At5g26680	+			FANCM	At1g35530	+	
	PARP1	At2g31320	+			EME1A	At2g21800	+	
	PARP2	At4g02390	+			EME1B	At2g22140	+	
	Pol δ	See section E				MUS81	At4g30870	+	
	Pol ϵ	See section E				GEN1	At1g01880	+	
	LIG1	See section E				SEND1	At3g48900	+	
**B**	Nucleotide excision repair (NER)					TOP3α	At5g63920	+	
	RAD4	At5g16630	+			RMI1	At5g63540	+	
	RAD23A	At1g16190	+			Pol δ	See section E		
	RAD23B	At1g79650	+	5		PCNA	See section E		
	RAD24C	At3g02540	+	5		RFC	See section E		
	RAD23D	At5g38470	+	5	**D**	DNA mismatch repair (MMR)			
	CEN2	At4g37010	+			MSH2	At3g18524	–	
	DDB1A	At4g05420	+			MSH3	At4g25540	–	
	DDB1B	At4g21100	–			MSH6	At4g02070	+	
	DDB2	At5g58760	+			MSH7	At3g24495	+	
	CSA	At1g27840	+			MLH1	At4g09140	+	
		At1g19750	+			RFC	See section E		
	CHR8	At2g18760	+			PCNA	See section E		
	CHR24	At5g63950	+			EXO1	See section E		
	XPB1	At5g41370	+			RPA	See section F		
	XPB2	At5g41360	+			POL δ	See section E		
	UVH6	At1g03190	+		**E**	Components involved in metabolic pathways			
	TFIIH1	At1g55750	+			EXO1	At1g29630	+	
		At1g61420	+			PCNA	At1g07370	–	
	GTF2H2	At1g05055	–				At2g29570	–	
	TFIIH3	At1g18340	+			Pol δ	At1g09815	–	
	TFIIH4	At4g17020	+				At2g42120	+	
	TTDA	At1g12400	+				At5g63960	+	
		At1g62886	–			Pol ϵ	At1g08260	–	
	CDKD;1	At1g73690	–				At2g27120	+	
	CDKD;2	At1g66750	+				At5g22110	+	
	CDKD;3	At1g18040	+			RFC	At1g21690	+	
	CYCH;1	At5g27620	+				At1g63160	–	
	MAT1	At4g30820	+				At1g77470	+	
	UVH3	At3g28030	+				At5g22010	+	
	UVH1	At5g41150	+	6			At5g27740	–	
	ERCC1	At3g05210	+			LIG1	At1g08130	–	
	RPA	See section F					At1g49250	–	
	PCNA	See section E			**F**	Replication protein A (RPAs)			
	RFC	See section E				RPA1	At2g06510	+	
	Pol δ	See section E					At5g08020	+	
	Pol ϵ	See section E					At5g45400	–	
	LIG1	See section E					At5g61000	–	
							At4g19130	–	
						RPA2	At2g24490	–	
							At3g02920	+	
						RPA3	At3g52630	+	
							At4g18590	+	

To estimate the extent of AS in DNA repair genes at the genome-wide level, we queried the Arabidopsis reference transcript dataset (AtRTD2), which contains 82,190 transcripts from 34,212 genes (Zhang et al., 2017b), with a list of 102 Arabidopsis DNA repair genes (Spampinato, 2017). Only nine genes from this list have previously been reported to be alternatively spliced. Remarkably, this survey revealed that more than 80% of these genes show evidence of AS in the AtRTD2 (**Table 1**). Further, key regulators of DDR in plants, SOG1, ATM, and ATR (not in the Spampinato, 2017 list), also undergo AS. Although this brief survey deals with a subset of DDR genes, it clearly illustrates a hidden potential for AS and regulation of DDR in plants. Plant mechanisms and SFs involved in DDR regulation remain to be investigated.

## Conclusions

The cellular response to DNA damage must be tightly regulated. Numerous studies on animals reveal interactions between DDR and AS at multiple levels and demonstrate that AS has an important role in DDR. In plants, initial studies show that AS has a function in plant DDR, but many questions remain to be addressed. How is the expression and activity of plant SFs regulated in DDR, what are their target genes, and do RNAPII processivity or changes in chromatin structure convey DDR into differential splicing outcomes in plants? Comprehensive transcriptome analyses will identify genes that show differences in AS patterns in response to genotoxic stress. Moreover, SFs, RNA processing factors, and DNA repair genes that undergo changes in AS may be detected and help determine the complex interplay between DDR and AS in plants. Finally, the major stress factors restrict plant growth and decrease yield in crop plants. Recent studies report extensive AS in crop species, emphasizing the need for further investigations to establish AS involvement in the response mechanisms to stress exposure and DNA damage.

## Author Contributions

MK designed the project. BN performed the survey of alternative splicing of Arabidopsis DNA repair genes and prepared the table and figure. The manuscript was written by BN, SR, and MK.

## Funding

This work is supported by the Austrian Science Fund (FWF) (P26333 to MK).

## Conflict of Interest

The authors declare that the research was conducted in the absence of any commercial or financial relationships that could be construed as a potential conflict of interest.
